# T-Cell Differentiation and Progression of HIV Infection

**DOI:** 10.1371/journal.pbio.0020041

**Published:** 2004-02-17

Of the 300 or so viruses that cause disease in humans, HIV may have the greatest adaptive advantage. Like most persistent viruses—including the herpesviruses Epstein–Barr and cytomegalovirus (CMV)—HIV employs various strategies to counteract its host's response to infection. But HIV possesses a unique ability to sustain a progressive attack on the immune system—infecting the very cells that coordinate the immune response—leaving the body susceptible even to normally harmless microorganisms. It is these so-called opportunistic infections, rather than the human immunodeficiency virus itself, that makes HIV so deadly. The specific mechanisms that engineer this ongoing systemic attack have been the subject of intense research.

HIV targets white blood cells with protein surface receptors called CD4. These CD4, or helper, T-cells normally orchestrate the body's immune response by signaling killer T-cells (which are also called CD8 T-cells, after their CD8 surface receptors) and other immune cells to multiply and differentiate—that is, become specially equipped to recognize a particular pathogen, or antigen. At the onset of infection, the immune system appears to respond normally, with a strong attack led by HIV-specific CD8 T-cells that initially contain the virus. But as the infection progresses, CD4 counts drop and the body's ability to renew T-cells decreases while its proportion of “antigen-experienced” CD8 T-cells increases. While the biological effect of this hyperactivity is unclear, it is apparent that patients with elevated immune activity face a poor prognosis. Investigating the interaction among immune activation, CD8 T-cell differentiation, and HIV prognosis, Victor Appay and colleagues report that a close connection between elevated immune activation and elevated levels of highly differentiated T-cells may bring further insights into how HIV exhausts the immune system.

To examine the effect of elevated immune activation on T-cells, the researchers analyzed T-cells from a group of HIV-infected individuals collected at two distinct points in time: at the onset of acute infection—which is characterized by vigorous HIV replication—and after treatment, when viral replication is suppressed. To explore the connection between T-cell differentiation and clinical status, the researchers analyzed the T-cells from a group of untreated infected individuals divided into three subsets based on stage of infection: acute infection, chronic infection without progression, and chronic infection with signs of progression.

During acute HIV infection, the vast majority (80%–90%) of the CD8 T-cell population was activated—not just the HIV-specific CD8 T-cells. Surprisingly, CD8 T-cells specific to the Epstein–Barr and CMV viruses showed significant activation levels during acute infection, suggesting that HIV may indirectly promote the replication of these viruses. When the researchers investigated the effects of this activation on T-cell differentiation, they found a correlation between increasing antigen concentrations and increasing CD8 T-cell activation and proliferation. And when Laura Papagno et al. analyzed the differentiation state of CD8 T-cells in individuals at different stages of infection, they found a progression in the proportion of highly differentiated CD8 T-cells associated with HIV disease progression.

These results, the researchers conclude, show that chronic overactivation of the immune system during HIV infection produces the large pool of highly differentiated T-cells observed in HIV infection. T-cells go through various stages toward late differentiation, and it may be that the early-differentiated CD8 T-cells, which maintain the ability to proliferate, offer protective immunity. But highly differentiated cells, they propose, exhibit characteristics associated with “replicative senescence”—they are in effect old, worn-out cells that can no longer proliferate. Though replicative senescence is a natural process for most cells, in the context of HIV—in which infected individuals also lose the ability to replenish T-cells—it creates an aging population of T-cells that are less effective at fighting infection.

**Figure:**
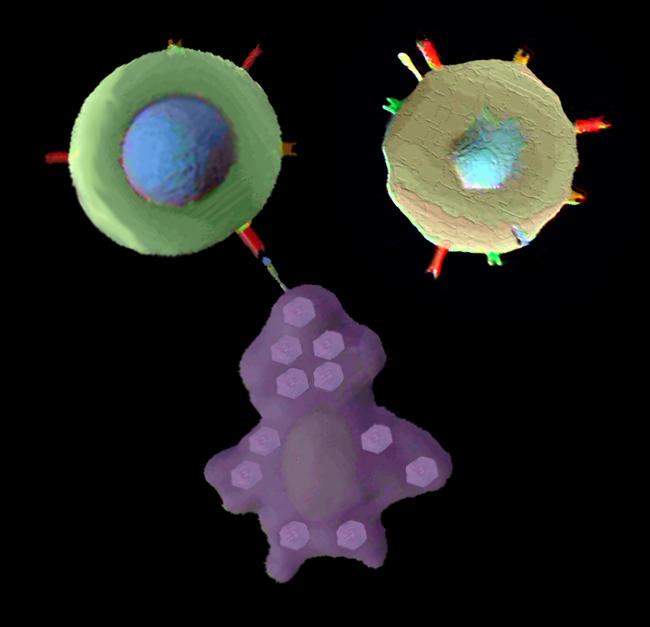
Two T-cells, one of which recognizes a target cell

